# Supplementary material for: Improving 3D deep learning segmentation with biophysically motivated cell synthesis
Source: Commun Biol. 2025 Jan 11;8:43. doi: 10.1038/s42003-025-07469-2 (PMC11724918; doi:10.1038/s42003-025-07469-2)
Supplement: Supplementary file 1 — Supplemental Material [file 42003_2025_7469_MOESM1_ESM.pdf]

Supplementary material:

Improving 3D deep learning segmentation with biophysically motivated cell synthesis

Roman Bruch[1*], Mario Vitacolonna[2,3], Elina Nürnberg[2,3], Simeon Sauer[2,4], Rüdiger Rudolf[2,3], Markus Reischl[1]

[1*]Institute for Automation and Applied Informatics, Karlsruhe Institute of Technology, Hermann-von-Helmholtz-Platz 1, Eggenstein-Leopoldshafen, 76344, Germany.
[2]Institute of Molecular and Cell Biology, Mannheim University of Applied Sciences, Paul-Wittsack-Straße 10, Mannheim, 68163, Germany.
[3]CeMOS, Mannheim University of Applied Sciences, Paul-Wittsack-Straße 10, Mannheim, 68163, Germany.
[4]CHARISMA, Mannheim University of Applied Sciences, Paul-Wittsack-Straße 10, Mannheim, 68163, Germany.

*Corresponding author(s). E-mail(s): roman.bruch@kit.edu;

Appendix A Overview of analyzed approaches for nuclei and membrane synthsesis

Figure A1 shows an overview of the tested transformation approaches for membrane and nuclei synthesis. The main objective was the generation of synthetic nuclei data for the training of segmentation models. We analyzed several ways of creating nuclei images based on the biophysical simulation of cell borders. One approach is based on placing nuclei into the simulated cell borders which is described as SimOptiGAN+ in the main paper. The other three approaches utilize a GAN-based transformation between membrane like signals and nuclei signals. These approaches are subdivided based on the model which enables the transformation. Two of them are described in the main paper: Mem2NucGAN-P which is based on the transformation model *Binary Membrane → Nuclei (Paired)* and Mem2NucGAN-U which is based on the transformation model *Binary Membrane → Nuclei (Unpaired)*. The third GAN-based

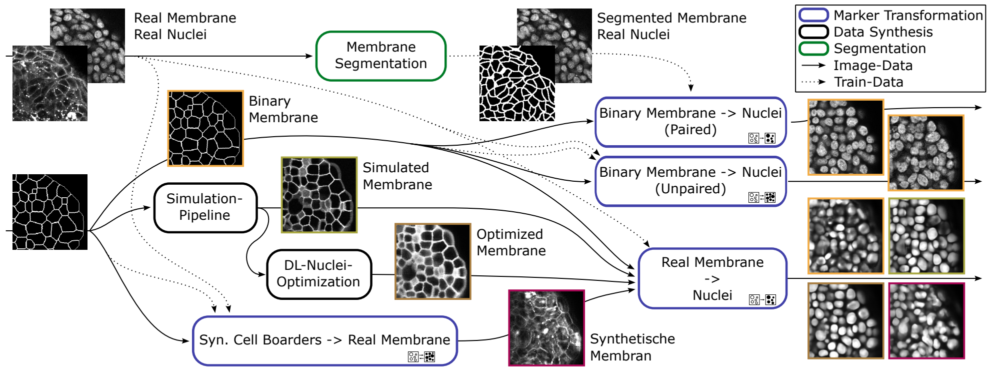

Fig. A1 Overview of methods for the transformation of synthetic cell borders to nuclei images.

transformation model *Real Membrane* $\rightarrow$ *Nuclei* is only described in the following, as generated nuclei signals lack texture. We, however, feel that this approach has the potential to generate realistic nuclei.

Previous experiments indicated that a conditional GAN can be used to transform real membrane signals into realistic nuclei signals. As this transformation is learned based on paired images of membrane and nuclei markers, the model can build the implicit knowledge about the correlation between membrane and nuclei morphology. Therefore, if such a trained model is used to transform synthetic membrane signals, realistic nuclei signals are expected. As the simulated cell borders are vastly different from real membrane signals, a direct transformation will most likely fail due to the domain gap to the training data. Therefore, different approaches are tested to reduce visual differences.

The first approach *Simulation-Pipeline*, utilizes the previously presented pipeline SimOptiGAN [1] to model optical effects introduced by the microscope. To be specific, only the imaging simulation part is used. During this part, the point spread function (PSF), brightness reduction and noise are modeled and applied to the cell border image. The resulting image *Simulated Membrane* is highlighted in green color.

The second approach uses the *Simulated Membrane* image and applies a GAN-based post-processing, as presented in [1]. This post-processing is trained based on unpaired images of simulated nuclei and real nuclei. The model can be applied to membrane signals, as it only performs a fine-tuning of the simulated nuclei. The resulting image *Optimized Membrane* is highlighted in brown color.

The third approach is the most sophisticated of the three. This relies on an additional GAN-based model to transform the synthetic cell labels into realistic looking membranes. This transformation is based on a CycleGAN utilizing unpaired data of simulated cell borders and real membrane signals for training. The trained model *Synthetic Cell Borders* $\rightarrow$ *Membrane* is then used to generate the image *Synthetic Membrane* highlighted in red. Each of the three pre-processed images is then used for the inference of the model *Real Membrane* $\rightarrow$ *Nuclei.* Additionally, the naive approach of directly transforming the binary membrane image is also shown (yellow color).

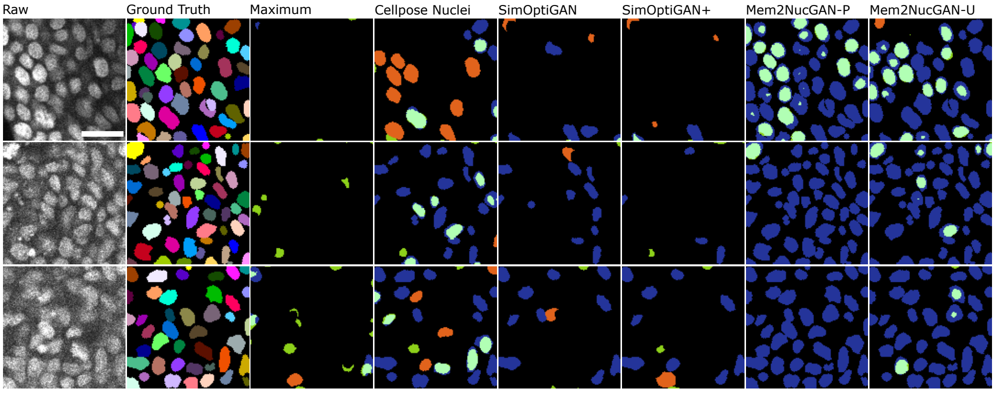

Fig. A2 Comparison of nuclei segmentation results. Only one 2D slice of each ground truth patch is visualized for enhanced clarity. The first and second column display the raw image signal and its corresponding ground truth, while subsequent columns show the errors made by the segmentation models regarding the DET measure, including false-positive (green), false-negative (blue), and required splitting operations (orange). Overlaps between false-positives and false-negative errors result in a light green color. Scale bar: $50\mu m$.

Appendix B Model training and inference details

The complete code used in this manuscript is available at the linked GitHub repository. In the corresponding README file, an instruction is given on how the analysis can be reproduced. Additionally, all data including raw image files, intermediate and final results, and parameter settings are available at Zenodo. In the following, a detailed description is given of the generation process of the synthetic data.

StarDist Models

The presented data synthesis methods were evaluated based on the segmentation performance of a 3D StarDist model. For training and inference, we utilized the provided example scripts from the StarDist repository[1]. In the training script, images were normalized, and small label holes were filled. Training data was then randomly divided into a train and validation set with a split of 0.85 to 0.15 but with at least one validation image. Key parameters were set as follows: *n_rays* was kept at the default value of 96, and a subsampled grid was used if the anisotropy exceeded 1.5, as in the example. The training patch size was $96 \times 256 \times 256\,\text{px}^3$ (z, y, x), and the training batch size was set to two. In cases where manual annotated patches were used for training, the training patch size had to be reduced to $32 \times 128 \times 128\,\text{px}^3$ (z, y, x) to match the size of the ground truth patches. Augmentation was applied using the provided functions for flipping, rotation, and intensity changes. Random seed settings were removed from the script to enable statistical analysis. All models were trained for 100 epochs. Post-training, thresholds (probability and non-maximum suppression) were optimized based on the validation data. For model inference, weights from the

[1] https://github.com/stardist/stardist

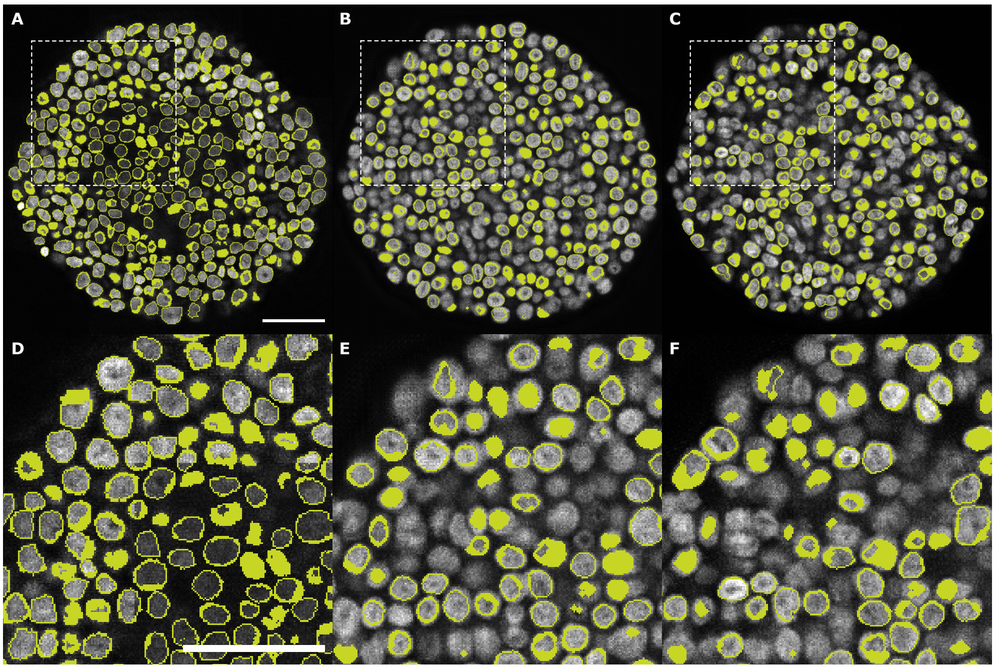

Fig. A3 **A-C** Image sections of generated nuclei signals and the corresponding labels as yellow boundary overlay (**A** SimOptiGAN, **B** Mem2NucGAN-P and **C** Mem2NucGAN-U). The dashed rectangle indicates the zoom region visualized in **D-F**. While the labels of SimOptiGAN+ show consistency with the image data, Mem2NucGAN-P and Mem2NucGAN-U seem to feature some inconsistencies with the image data. It should be noted, that for these two methods, the labels are more compact, especially in the z-axis. This might lead to the impression, that some nuclei miss a label, which, however, just gets visible in the neighboring slice. Scale bars: $50\mu m$.

epoch with the best validation loss were selected, and the image data was normalized as in the example script before processing.

SimOptiGAN

With SimOptiGAN a total of five images were generated. The corresponding parameter files can be found in the data repository. The implementation of the optimization model is based on the official implementation of the CycleGAN. The optimization model was trained with five simulated images and one large real image. During training, patches of size $32 \times 256 \times 256\,\text{px}^3$ (z, y, x) were randomly extracted from the training images. The model was trained for 3000 epochs with a constant learning rate of 2×10^{-4}, followed by an additional 3000 epochs with a linear decreasing learning rate. Notably, the dataset size was virtually doubled to address performance issues related to model performance tracking, effectively doubling the number of samples processed per epoch. Intermediate results were saved, and the epoch yielding the most visually satisfactory results (epoch 2994) was selected. Inference was performed on the simulated training data using a patch-based approach, with the same patch size used in training and a 50% overlap. Overlapping regions were cropped from transformed

patches before image reassembling. Subsequent segmentation training was conducted using four optimized images.

SimOptiGAN+

SimOptiGAN+ builds upon the results of biophysical simulations, specifically utilizing simulated cell boundary images. For each of the four cell boundary images, a synthetic nuclei image was generated using SimOptiGAN+. The corresponding parameter files are available in the data repository. The optimization process employed the previously trained SimOptiGAN model (see the preceding paragraph for details). Subsequent segmentation training was again carried out using the four optimized images.

Mem2NucGAN-P

Mem2NucGAN-P requires segmented real membrane images for training of the cGAN based transformation model. The segmentation of these images was performed using the pretrained Cellpose cyto2 model, with the parameters `diameter` and `stitch_threshold` set to 22 and 0.5, respectively. Remaining parameters were left untouched. Additionally, Mem2NucGAN-P requires unpaired binary segmentation masks of nuclei. For this, a previously introduced segmentation model [1], trained on synthetic data, was used. Four simulated images, four real binary membrane segmentations and four binary nuclei segmentations were then used to train the cGAN transformation model. The implementation of the cGAN is based on the pix2pix implementation, but utilized a ResNet as generator model. The cGAN was trained for 1400 epochs with a constant learning rate of 2×10^{-4}, followed by 1400 epochs with a linear decreasing learning rate. In this case, the final epoch (1400) was selected for further calculations. Like for the optimization model of SimOptiGAN, the dataset size was virtually increased by a factor of four, which leads to a similar number of seen samples per training (11200-Mem2NucGAN-P to 12000-SimOptiGAN). The implementation employed the same patch-based training and inference approach as used in SimOptiGAN optimization. Using the same four biophysically simulated cell boundary images as in SimOptiGAN+, the trained cGAN generated four synthetic images containing synthetic nuclei signals and corresponding synthetic labels. A post-processing step was then performed to convert the network's output into instance labels. Subsequent segmentation training was conducted using these four generated images and their corresponding instance labels.

Mem2NucGAN-U

Mem2NucGAN-U employs a CycleGAN model for data generation, allowing the use of unpaired images during training. This capability enables the direct utilization of biophysically simulated cell boundary images in combination with real images and unpaired binary nuclei segmentations as training data. For training the CycleGAN, four biophysically simulated cell boundary images (the same as those used in Mem2NucGAN-P inference), along with four real images and four binary nuclei segmentations (the same as those used in Mem2NucGAN-P training), were utilized. The model was trained for 1400 epochs with a constant learning rate of 2×10^{-4} and 1400 epochs with a linear decreasing learning rate, with the final epoch (1400) selected for

further calculations. As four the other GAN based models, the dataset size was virtually increased by a factor of four, ensuring a similar number of samples were processed during the training. The same patch-based training and inference approach as used in SimOptiGAN optimization was applied here. Using the same four biophysically simulated cell boundary images employed during training, the trained CycleGAN was used to generate four synthetic images containing synthetic nuclei signals and corresponding synthetic labels. A post-processing step was then conducted to convert the network's output into instance labels. Subsequent segmentation training was carried out using these four generated images and their corresponding instance labels.

Appendix C Ablation Study Additional Discriminator

To analyze the importance of the introduced discriminator, which is responsible for enforcing a realistic segmentation in both Mem2NucGAN methods, an ablation study was conducted for the CycleGAN based Mem2NucGAN-U method. It is important to note that the additional discriminator is the sole structure that ensures a realistic looking segmentation and a high contrast between foreground and background signals. The omission of this discriminator resulted in a segmentation output that closely resembled the input signal, albeit with a drastically reduced contrast. Additionally, the training process appeared to be more unstable. One potential explanation for this is the smaller loss term associated with the generator model. Given that, apart from the final convolution, the path of both output channels is identical, the loss term of the additional discriminator exerts influence over the majority of the network parameter set.

It is also important to note that identity loss cannot be used to achieve high-contrast segmentation outputs, as the number of input and output channels differs between the two generators. Prior to the introduction of the additional discriminator, we tested a difference loss (L1) between the generated image (constant) and the generated segmentation signal, with the aim of enforcing a matching between the two signals in combination with a loss that enforces a binary-like output:

$$\mathcal{L}_{seg}(G_{A2B}) = \mathbb{E}_{xy\sim p_{data}(x)}\left[\,\|G(x)_{img} - G(x)_{seg}\|_1\,\right] + \mathbb{E}_{x\sim p_{data}(x)}\left[\,\|bin(G(x)_{seg}) - G(x)_{seg}\|_2\,\right]. \quad \text{(C1)}$$

However, that training structure was less stable and yielded inferior results compared to the additional discriminator.

References

[1] Bruch R, Keller F, Böhland M, et al (2023) Synthesis of large scale 3D microscopic images of 3D cell cultures for training and benchmarking. PLOS ONE 18(3):e0283828

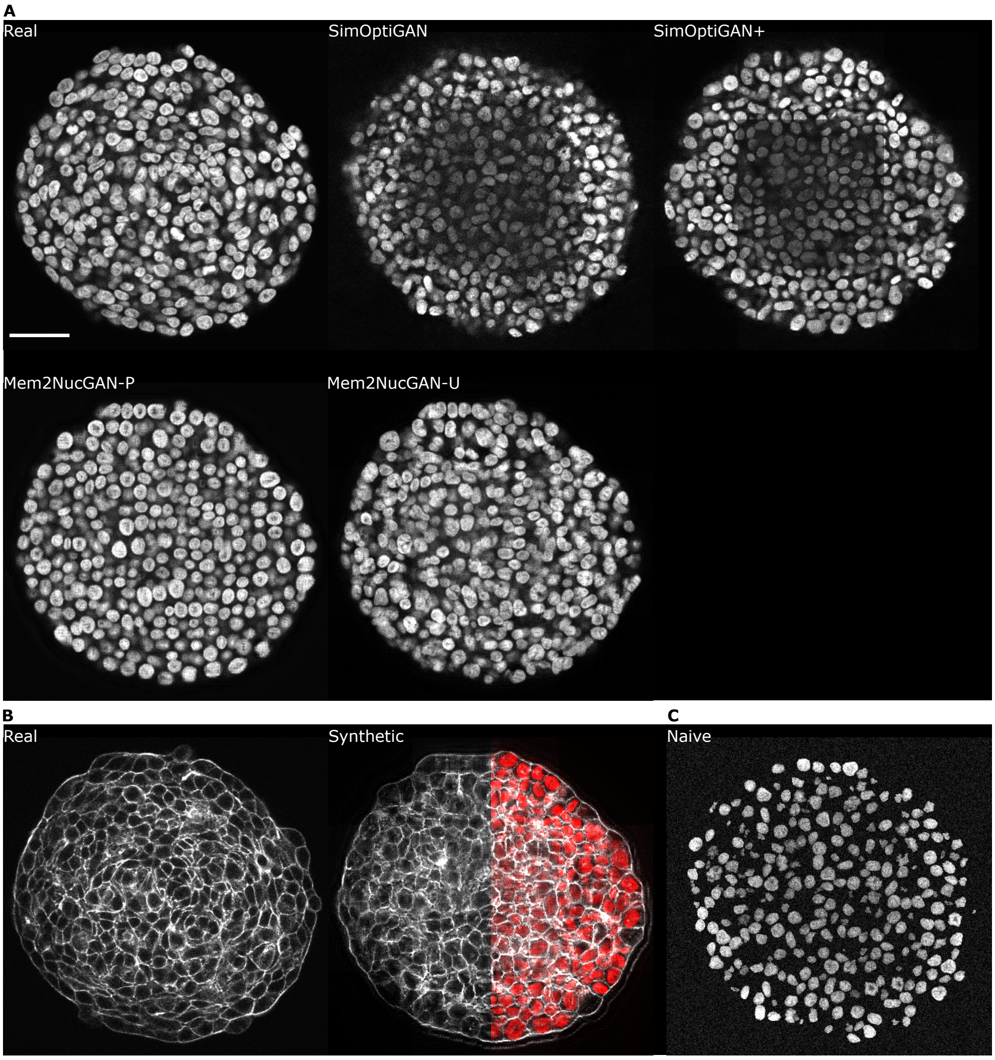

Fig. A4 Detailed view of the images presented in Fig.4 of the main article. **A** image slices of real and synthetic 3D nuclei images. **B** image slices of real and synthetic membrane signals. The membrane signal exhibited a consistent cell arrangement compared to SimOptiGAN+, Mem2NucGAN-P, and Mem2NucGAN-U, as demonstrated by the overlay of synthetic membrane with nuclei generated using SimOptiGAN+. **C** provides a preview of naively generated data, which serves as the worst-case example for the KID evaluation. Scale bar: $50\mu m$.
